# A virtual bird’s eye view: Live streaming nest boxes to continue outreach in the era of COVID‐19

**DOI:** 10.1002/ece3.6998

**Published:** 2020-11-20

**Authors:** Jennifer L. Houtz, Rachael P. Mady, Jennifer J. Uehling

**Affiliations:** ^1^ Department of Ecology and Evolutionary Biology Cornell University Ithaca NY USA; ^2^ Cornell Lab of Ornithology Ithaca NY USA; ^3^ Department of Natural Resources Cornell University Ithaca NY USA

**Keywords:** cavity nesters, live stream, nest box, science outreach

## Abstract

COVID‐19 created a host of challenges for science education; in our case, the pandemic halted our in‐person elementary school outreach project on bird biology. This project was designed as a year‐long program to teach fifth‐grade students in Ithaca, New York, USA, about bird ecology and biodiversity using in‐person presentations, games, activities, and outdoor demonstrations. As a central part of this effort, we set up nest boxes on school property and planned to monitor them with students during bird breeding in the spring. Here, we describe our experiences transitioning this program online: we live streamed nest boxes to the students’ virtual classroom and used them as a focal point for virtual lessons on bird breeding and nestling development. In an era of social distancing and isolation, we propose that nest box live streaming and virtual lessons can support communities by providing access to the outdoors and unconventional science learning opportunities for all students. Instituting similar programs at local schools has the potential to increase equitable learning opportunities for students across geographic locations and with varying degrees of physical access to the outdoors and nature.

## Graphical abstract

COVID‐19 created a host of challenges for science education, including public outreach. Here, we describe our experiences transitioning a fifth‐grade outreach program on bird biology online, and specifically focus on our live streaming of nest boxes as a starting point for virtual lessons on bird breeding and nestling development. We suggest that such virtual programs can promote equitable learning opportunities for students across geographic locations and with varying degrees of physical access to the outdoors and nature, supporting communities through a difficult era of social distancing and isolation.

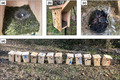

## 1 INTRODUCTION

While the COVID‐19 shutdown and associated in‐person school cancellations created significant limitations for science education, it simultaneously created opportunities for scientists to develop new methods for outreach to the general public via virtual platforms. Here, we share our perspective on transitioning a bird ecology and biodiversity outreach project online to provide insight for others who may be transitioning outreach programs to virtual formats. We initially created this outreach program for fifth graders with the goals of increasing general knowledge about birds and biodiversity, fostering long‐term appreciation for nature, and introducing field ecology as a potential career option to young students. Prior to the suspension of in‐person classes due to COVID‐19, we set up nest boxes at a local elementary school as a teaching tool for this outreach program and had been using them as a springboard for lessons about bird biology and behavior. In addition to providing teaching opportunities, these nest boxes also provided breeding habitat for cavity‐nesting birds. After the school's instruction transitioned online, we continued to visit the fifth‐grade classrooms virtually. We found that the nest boxes were an ideal focal point for virtual instruction, allowing us to teach the students about bird breeding and biology and take them on virtual “field trips” to check on the status of the boxes and the birds that occupied them.

Nest boxes can facilitate a transition from in‐person to virtual science outreach or the initiation of a virtual outreach project. They can be placed in almost any outdoor community space and can be easily filmed with an attached or mobile camera, including those found on smartphones. Virtual community outreach projects can not only make access to science education and nature more equitable for all students, but also promote a sense of community during a time when opportunities for connectedness are limited. Below, we describe our process of transitioning outreach to a virtual format and discuss how virtual outreach may promote more equitable outdoor education opportunities. We encourage others to consider similar courses of action to support their communities during COVID‐19 and beyond.

## 2 PROJECT INITIATION AND INTERRUPTION FROM COVID‐19

We started the fifth‐grade outreach program “A Bird's Eye View” four years ago as part of a volunteer program for Cornell graduate students: Cornell Graduate Student School Outreach Program (GRASSHOPR). Through the program, we were matched with the fifth‐grade teachers at Belle Sherman Elementary School in Ithaca, New York, USA. Every spring for three years, we taught a minicourse about birds for fifth graders and through this process built our relationship with the teachers. In the fourth year of outreach, with the teachers’ support, we decided to expand the program outside of GRASSHOPR by adding fieldwork components, planning visits throughout the entire school year, and working to establish student engagement in and excitement about bird habitat creation.

As part of our new and expanded “A Bird's Eye View” program in the 2019–2020 school year, we planned visits to Belle Sherman across the academic year so that we could teach students about birds’ annual cycles and maintain a connection with them over the course of their entire fifth‐grade year. As we planned our lessons, we focused on establishing a sense of connection between the students and the birds around their school. Belle Sherman is located next to open fields and a small wooded area with a short nature trail. As scientists who study cavity nesters, we realized that this could be excellent habitat for nest boxes to attract cavity‐nesting birds such as tree swallows (*Tachycineta bicolor*), black‐capped chickadees (*Poecile atricapillus*), Eastern bluebirds (*Sialis sialis*), and house wrens (*Troglodytes aedon*). Nest boxes are commonly used by researchers to study these species (Brennan et al., 1999; Vitousek et al., 2018; Willner et al., 1983) and are frequently used by the general public to attract cavity‐nesting birds to their properties. We designed lesson plans around exploring local bird diversity on and near Belle Sherman's campus and setting up nest boxes for the students and teachers to monitor.

**Figure 1:**
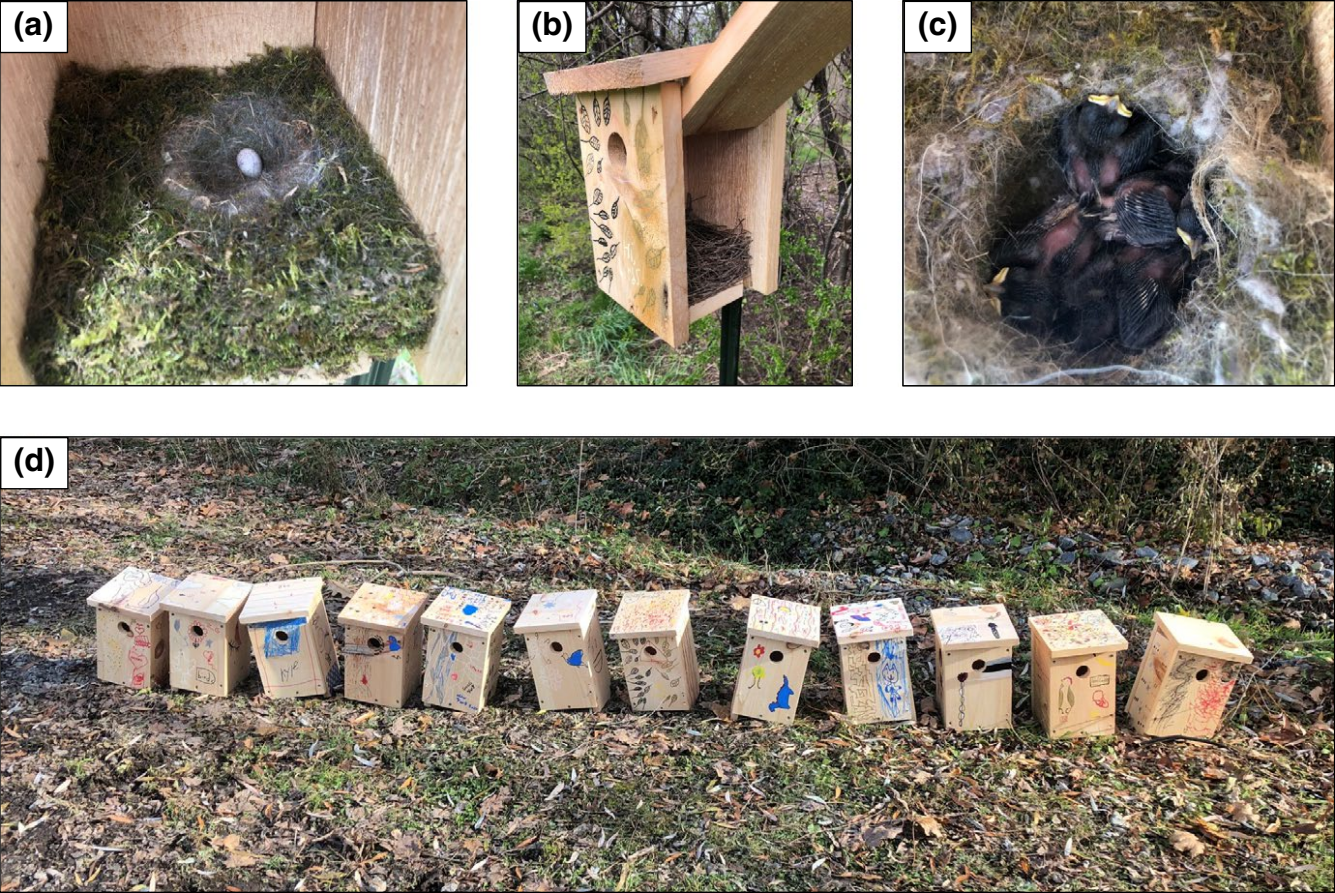
(a) A black‐capped chickadee (*Poecile atricapillus*) egg in one of the nest boxes set up at Belle Sherman Elementary in Ithaca, New York, USA. (b) An opened nest box to show the Eastern bluebird (*Sialis sialis*) nest built within. (c) Black‐capped chickadee nestlings inside a nest box. (d) Twelve nest boxes decorated by Belle Sherman fifth graders prior to installation on poles next to the elementary school

We taught four in‐person lessons over the course of the fall and winter. Our first two lessons focused on exploring bird biodiversity, ecology, and field methods. During these lessons, we showed students gear used by ornithologists to study birds, such as mist‐nets, banding pliers, leg bands, and measurement tools. We also used mist‐nets to capture birds near the wooded area and demonstrated bird handling and banding techniques. In our third lesson, we taught the students about bird annual cycles and nests and had students decorate premade nest boxes with permanent markers as a way to increase student involvement and interest in the project (Figure 1). We set up these nest boxes next to the school's campus, situating them along the edge of the school's fields and in the woods by the aforementioned trail. In our fourth lesson, we taught students about migration, aiming to get them excited about the migratory birds that would soon be returning to Ithaca while also describing the difficulties of migration, especially those caused by human activity and development (e.g., building collisions). As part of this lesson, we played a migration game during which students rolled dice to determine their fates as they navigated the many challenges of their mock “migration” across the classroom. For example, based on the numbers they rolled, some students were blown off course or eaten by outdoor cats, whereas others successfully completed their migrations and raised nestlings. Unfortunately, this migration lesson marked our final in‐person visit because the COVID‐19 shutdown and full transition to online learning occurred shortly thereafter.

## 3 TRANSITION TO VIRTUAL OUTREACH

Although there were no expectations to continue the program, we were inspired by the efforts of different organizations to adapt to virtual outreach and education, both pre‐COVID‐19 and during the pandemic. For example, the Cornell Lab of Ornithology developed education materials around their online Bird Cams (Fee et al., 2015), allowing teachers and students to connect with nature virtually. Using our smartphones, we could similarly live stream the nest boxes by Belle Sherman's campus as a virtual “field trip.”

With the nest boxes as the anchor, we taught two virtual lessons on bird breeding and nestling development. Both lessons incorporated live‐streamed “field trips” to check the contents of the nest boxes; however, we still needed to find ways to engage the students in learning about biology over a digital platform, beyond simply showing them live footage. We could no longer use “think‐pair‐share” (Lyman, 1981) or activities such as our migration game that involved students physically moving around the classroom, so we explored other avenues for engagement. Inspired by online question and answer webinars, for each lesson, we shared a Google Form with the students ahead of time to give them the opportunity to engage with material beforehand, ask questions, and share their thoughts.

In our Google Form for the breeding birds lesson, students were asked to watch a video of an American robin (*Turdus migratorius*) from the Cornell Lab Bird Cams (“Life on the Ledge: 2017 American Robin Cam Highlights”) depicting different stages of the breeding cycle. Students submitted questions about adult and nestling behaviors such as fecal sac removal, nestling begging responses, and feeding rates. We next asked students to guess which species of cavity‐nesting bird they thought nested in the Belle Sherman nest boxes, given the choices of black‐capped chickadee, European starling (*Sturnus vulgaris*), Eastern bluebird, and tree swallow.

In our Google Form for the nestling development lesson, students were instructed to watch a video of an adult barred owl (*Strix varia*) feeding its nestlings (“From Box to Branch: 2017 Barred Owl Cam Highlights”). We then asked the students to guess which food items are consumed by nestling owls versus black‐capped chickadees, given the choices of worms, flying insects, other birds, squirrels, and berries. Students were also given the opportunity to ask any questions they had via the Google Form; they asked questions about sibling competition, mass gain, and the role of adult males in nestling care.

After collecting responses and questions via the Google Forms and preparing material to share virtually, it was time for us to teach the students. We conducted each virtual lesson in Google Meet, the platform the teachers were already using to teach the students. For the first lesson, RPM and JJU joined via the cameras on computers in their own homes, and JLH, who was in the field near the nest boxes, joined via the camera on her personal smartphone, using her personal data plan to access the meeting. For the second lesson, JLH and JJU both joined the meeting via personal smartphone cameras from the field. We began each virtual lesson with a Google Slides presentation that included key terminology (e.g., altricial vs. precocial) and answers to questions that students had submitted on the Google Form beforehand. We then instructed the students to pin JLH’s or JJU’s video before we began the “virtual field trip” portion of the lesson, and JLH or JJU used their personal smartphone cameras to show the inside of the nest boxes. Of the twelve nest boxes we installed, two were used by black‐capped chickadees, two by house wrens, and one by Eastern bluebirds. With the timing of our lessons, we were able to live stream video of chickadee eggs and nestlings as well as house wren eggs. These nests gave us the opportunity to discuss how to identify different species based on nesting material and egg color with the students.

After live streaming the nest box contents, we reinforced the material at the end of each lesson with a “which bird are you” quiz for the breeding birds lesson and a dietary strategy guessing game for the nestling development lesson. Students actively participated by writing answers down and sharing their results in the Google Meet chat window or holding up their answers drawn on paper to their computers’ cameras, and we were able to summarize and communicate bird breeding biology and species differences in an interactive way.

In addition to their presubmitted questions, students could ask on‐the‐spot questions during the live stream of the nest boxes and access the presentation slides afterward. We found that by allowing students to engage with the material ahead of, during, and after the lesson, we could accommodate different learning styles (Campbell et al., 1996). Previous work has found that online asynchronous discussions encourage students who may not have participated in a classroom to engage with the material (Comer & Lenaghan, 2013). We also suspect that by incorporating asynchronous material like the Google Form beforehand and presentation slides afterward, students unable to attend the live lessons were still able to learn this material at a different time.

## 4 LESSONS LEARNED AND FUTURE STEPS

In adapting our in‐person lessons to be virtual, we realized that nest box monitoring (a) offers a flexible ecological teaching tool for educators and (b) creates opportunities for all students to engage with nature, regardless of their ability to go outdoors in person.

### 4.1 Nest boxes as flexible ecological teaching tools

Nest boxes are a low‐maintenance, interactive teaching tool that can be used in‐person or virtually to engage students in learning about birds and nature. Though we are trained ornithologists with federal and state permits, teachers and students do not need any permits to observe birds that nest in boxes (i.e., permits are only needed when handling or banding birds). Additionally, there are active community science (also known as citizen science) projects that provide instructions online for nest box placement, installation, and monitoring. These community science projects either provide instructions for how to build your own nest box (e.g., Cornell Lab of Ornithology's NestWatch program) or supply participants with prebuilt boxes for free or a small fee (e.g., Hawk Mountain Sanctuary Adopt‐a‐Kestrel Nest Box Program).

When using nest boxes as a teaching tool, we recommend educators set up multiple boxes to increase the probability of birds using at least one nest box. In all likelihood, not all nest boxes will be occupied. For example, we set up twelve nest boxes and birds only built nests in five out of twelve (42% occupancy rate). Educators can also take care to place boxes within the habitat requirements for species of interest in order to increase likelihood of occupancy. Finally, educators can increase the probability of nests successfully fledging (i.e., nestlings fully developing and leaving the nest) by installing predator guards (Bailey & Bonter, 2017), which are structures placed around the poles on which the nest boxes are mounted to deter predators from climbing up the poles into the boxes. For information about species’ habitat requirements, we recommend visiting the Cornell Lab of Ornithology's All About Birds website (www.allaboutbirds.org), and for more information about predator guards and ways to deter predators, we recommend visiting the Cornell Lab of Ornithology's NestWatch website (www.nestwatch.org).

In our program, we used nest boxes to teach about bird life cycles, bird breeding, and nestling development, but nest boxes can be used to teach lessons on a myriad of topics, including species identification, invasive species management (e.g., house sparrows (*Passer domesticus*) and European starlings), habitat selection, and behavior. Nest boxes can be used as teaching tools anywhere that cavity nesters live, meaning that lessons could be constructed around species in many different localities. For example, there are approximately 85 species of cavity‐nesting North American birds (Scott, Evans, Patton, & Stone, 1977), providing different options across the continent.

### 4.2 Live streaming nest boxes promotes equitable learning opportunities among students

Live streaming active bird nests may help all students access nature, regardless of the specific COVID‐19 restrictions in place in their regions or of their specific home situations. Live streaming does not require specialized cameras or wildlife research equipment and can be done with personal smartphones using existing internet connections, Wi‐Fi hotspots, or cellular data. The COVID‐19 pandemic has universally impacted the daily routines of people from all geographic locations and socioeconomic backgrounds, but to different degrees, making some outdoor activities such as bird watching more or less accessible. Here, we suggest that live streaming nest boxes and teaching lessons centered around these virtual field trips may help give all students, regardless of their specific geographic locations or living situations, the opportunity to experience nature and learn about bird biology.

In Ithaca, where the elementary school is located, playgrounds and children's gardens were closed for many months due to COVID‐19, making it more challenging for children to spend time outside in public places. Despite these closures, Ithaca is a rural college town in upstate New York surrounded by natural areas, and there are still many outdoor spaces available for socially‐distanced use. We recognize that limitations on outdoor activities are more severe in urban areas.

Before COVID‐19 restrictions were put in place, children from urban communities already had less physical contact with nature than those from rural communities. Children in urban areas are at higher risk for “extinction of experience,” whereby a lack of physical contact and/or emotional connection with nature causes neutral or negative attitudes toward ecological issues (Pyle, 1993; Soga & Gaston, 2016). COVID‐19 has likely further restricted urban children's access to the outdoors; cities have denser populations, making it harder to go outside without risking contact with others. Therefore, although we implemented our program in a rural region, we recommend virtual field trips using nest boxes as one possible solution to the lack of nature access for urban students. All students can participate regardless of their geographic location, promoting equitable opportunities for students to learn about biology and build experience with and appreciation for nature.

In addition to exacerbating outdoor access disparities between urban and rural students, COVID‐19 may also make it harder for some students to access the outdoors based on their living situations. Students likely face varying abilities to leave their homes due to guardian responsibilities or family health issues. NestWatch's nest box monitoring code of conduct suggests that children should always be accompanied by an adult when observing bird nests (“How to NestWatch”). This creates inequitable access to the outdoors among young students because some may have guardians whose work hours are such that they are unable to take their children outside. Additionally, some students may be completely unable to leave their homes because they must protect family members who are at a high health risk, or because they themselves are at a high health risk. Virtual nest box visits provide students, regardless of their specific living situations, the opportunity to see wild birds and connect to nature.

Even after COVID‐19 becomes a lesser threat, these virtual learning experiences will still provide avenues for equitable learning opportunities for students. For example, students living in urban regions will still have less access to green space and nature experiences compared to those living in rural regions, and virtual lessons with live‐streamed nest boxes could continue to provide important exposure to nature for all students.

## 5 LIMITATIONS

We have highlighted the benefits of using nest boxes as teaching tools during the COVID‐19 pandemic and beyond, but we recognize that this form of virtual outreach has limitations. Virtual instruction creates inequities in and of itself. In order for students to access or participate in virtual lessons, they must have reliable Internet access and a computer. When social distancing restrictions are lifted, these barriers could be addressed in some communities by encouraging students to go to their local libraries. Funding could also be allocated so that schools could provide each student with a portable computer to take home with them.

## 6 CALL TO ACTION

We call on local institutions and research groups to consider establishing partnerships with local elementary schools, following a virtual engagement plan similar to what we described above. Virtual field trips increase access to the outdoors regardless of social distancing restrictions. If such a partnership between local universities and elementary schools is not feasible, we encourage those who teach children to explore nest box monitoring as a teaching tool. Nationwide nest box monitoring programs such as NestWatch allow the general public to contribute to breeding bird surveys through community science data collection. Teachers, local conservation organizations, or parents/guardians can install nest boxes at their private residences or properties to create this type of program without explicit partnership from research institutions. The pandemic has made interactions with nature and each other difficult, and we recommend virtual nest box monitoring as a way for scientists and educators to support their local communities and promote connectedness between people and nature during this socially distanced era.

## CONFLICT OF INTEREST

The authors declare no competing interests.

## AUTHOR CONTRIBUTION

**Jennifer Lyn Houtz:** Conceptualization (equal); Funding acquisition (equal); Methodology (equal); Project administration (equal); Writing‐original draft (equal); Writing‐review & editing (equal). **Rachael Patricia Mady:** Conceptualization (equal); Funding acquisition (equal); Methodology (equal); Project administration (equal); Writing‐original draft (equal); Writing‐review & editing (equal). **Jennifer Jean Uehling:** Conceptualization (equal); Funding acquisition (equal); Methodology (equal); Project administration (equal); Writing‐original draft (equal); Writing‐review & editing (equal).

## Data Availability

We do not present any data in this paper.
